# Effects of face masks on speech recognition in multi-talker babble noise

**DOI:** 10.1371/journal.pone.0246842

**Published:** 2021-02-24

**Authors:** Joseph C. Toscano, Cheyenne M. Toscano

**Affiliations:** Department of Psychological and Brain Sciences, Villanova University, Villanova, PA, United States of America; University of California, Los Angeles, UNITED STATES

## Abstract

Face masks are an important tool for preventing the spread of COVID-19. However, it is unclear how different types of masks affect speech recognition in different levels of background noise. To address this, we investigated the effects of four masks (a surgical mask, N95 respirator, and two cloth masks) on recognition of spoken sentences in multi-talker babble. In low levels of background noise, masks had little to no effect, with no more than a 5.5% decrease in mean accuracy compared to a no-mask condition. In high levels of noise, mean accuracy was 2.8-18.2% lower than the no-mask condition, but the surgical mask continued to show no significant difference. The results demonstrate that different types of masks generally yield similar accuracy in low levels of background noise, but differences between masks become more apparent in high levels of noise.

## Introduction

Human speech perception is remarkably robust across a wide range of contexts. Listeners with normal hearing can understand speech even in relatively high levels of background noise [1], and they can cope with considerable acoustic variability between talkers’ voices [2, 3]. They can even recognize speech when presented with novel listening conditions, such as someone talking with a pen in their mouth [4].

One novel context is understanding speech produced while wearing a face mask. As a result of the COVID-19 pandemic, public health officials have recommended that individuals wear masks to help reduce the spread of the SARS-CoV-2 virus [5]. Masks are effective in decreasing transmission of the disease [6], and practices such as universal masking and social distancing have the potential to save many lives [7].

A potential concern with the use of face masks is that they may cause a reduction in speech intelligibility. We addressed this concern by investigating the effects of different types of masks on speech recognition in the context of multi-talker babble noise. We examined the effects of two homemade cloth masks, a surgical mask, and an N95 respirator, comparing performance to speech produced without a mask under conditions of both high and low levels of background noise.

Previous work has shown that face masks primarily attenuate sounds above 1 kHz, and different types of masks (e.g., N95 respirators vs. cloth masks) affect the speech signal to different degrees, in terms of both attenuation of high-frequency sounds [8, 9] and effects on the directivity of the signal [10]. Previous studies have also investigated perceptual effects of face masks, but the results have been mixed. Studies investigating effects of surgical masks and respirators used in healthcare settings have found little to no effect of surgical masks by themselves [11–13], while N95 and other respirators have variable effects (1-17% decrease in speech recognition accuracy; [14]). Other work has investigated fabric face coverings, finding a range of different results. Some studies find that these types of masks have little effect on speech recognition, beyond deficits associated with the loss of visual information [15], others find similar effects to other types of masks [16], and others find larger effects compared with other types of masks in reverberant environments, such as classrooms [17]. However, many of these studies had limited sample sizes, did not use pre-recorded materials, or experienced ceiling effects due to low levels of background noise.

The current study assesses the effects of speech produced while wearing a mask, which includes effects caused by the masks themselves (e.g., dampening of certain acoustic frequencies), as well as potential differences in how talkers produce speech as a consequence of wearing a mask. The specific context evaluated here involves conditions in which no visual cues are available, with recordings made using a microphone at close distance and sentences normalized to have the same average intensity. Although this differs from in-person face-to-face communication in important ways, it is directly applicable to contexts in which a talker may need to use a microphone while wearing a mask (e.g., while teaching), and it provides information about how the type of mask and level of background noise affect speech communication.

Listeners (N = 181) heard sentences selected from the Hearing in Noise Test [1] produced by two talkers (the two authors; one female [Talker 1, CMT], one male [Talker 2, JCT]; both native speakers of American English). Sentences were recorded while wearing no mask, a surgical mask, a homemade cloth mask with a fitted design, a homemade cloth mask with a pleated design, or an N95 respirator. Six-talker babble noise was added to the recordings at high (+13 dB) and low (+3 dB) signal-to-noise ratios (SNR). We measured the proportion of words in each sentence that listeners correctly recognized to assess the effect of each type of mask on speech recognition.

## Method

A pre-registration report summarizing the study design and analyses is available at: https://aspredicted.org/2yx96.pdf.

### Design

The study is a 2 (SNR; +3 vs. +13 dB) × 2 (talker; Talker 1 vs. Talker 2) × 5 (mask type; disposable surgical mask, fitted/center-seam cloth mask, pleated cloth mask, N95 respirator) experiment. Stimuli consist of recordings from two lists from the Hearing in Noise Test (HINT, Lists 3 and 19; [1]) embedded in six-talker babble noise. Listeners heard one sentence in each of the 20 experimental conditions. Forty trial lists were created as follows. Each HINT list includes 10 phonetically-balanced sentences, which were divided among the 10 mask × talker conditions in a Latin square design. One list was presented at each SNR, for a total of 20 unique trials/sentences for each subject. Stimulus presentation was also blocked by SNR; half of the subjects heard stimuli with the low SNR first and half heard stimuli with the high SNR first. Within each block, stimuli were presented in random order. The experiment took an average of 14 minutes to complete.

### Participants

A total of 200 subjects (73 female; mean age: 37 years old) participated in the study. This sample size corresponds to approximately 77 observations per parameter in our statistical analyses (4000 observations; 52 parameters in the model). Subjects were recruited using Amazon.com’s (Seattle, WA) Mechanical Turk service to obtain a sufficiently large sample, provided informed consent, and received monetary compensation for their time. The study was approved by the Villanova University Institutional Review Board.

### Stimuli

Sentences were recorded by two talkers (the two authors) using a Rode (Sydney, Australia) NT1 condenser microphone attached to a boom arm and PreSonus (Baton Rouge, LA) AudioBox USB audio interface in a quiet home office. Recordings were made with the microphone positioned approximately 6–8 cm in front of and adjacent to the left side of the talker’s mouth, were digitized at a sampling rate of 44.1 kHz with a bit depth of 24, and were and saved to computer for editing offline. Subsequently, we recorded the same sentences produced by the same talkers in a sound-attenuated booth, using the same microphone and a Focusrite (High Wycombe, UK) Scarlett 18i8 audio interface. The microphone was placed 7 cm from the left corner of the talker’s mouth, measured with a digital caliper. In addition, sentences were recorded twice, with masks worn in the opposite order, in order to counterbalance any effects of vocal fatigue. Acoustic analyses of these materials revealed similar patterns to those observed for the stimuli used in the experiment (see Supporting information).

All edits were made using Praat [18]. Raw recordings were first spliced into separate sound files for individual sentences. Spectrograms for each sentence were visually inspected to identify and remove artifacts (e.g., pops, clicks, non-speech mouth movements). Sounds were cut at zero-crossings or as close as possible to zero to avoid introducing any acoustic artifacts. Sentences were then normalized individually using the Scale Intensity function in Praat, so that they had the same average intensity, and the resulting sounds were resampled to 22.05 kHz to match the sampling rate of the multi-talker babble noise. This yielded a total of 200 sound files across the five mask types, two talkers, and 20 HINT sentences.

A recording of six-talker babble was used to generate background noise. Recordings for the babble noise came from the Wildcat Corpus [19], and consisted of semantically normal sentences spoken by three female and three male talkers (all native speakers of American English). From the original 50 second recording, periods of noise were randomly sampled, cutting sounds as closely as possible to zero-crossings. The length of each noise segment was approximately 2,500 ms, which was longer than the longest sentence recording (the longest sentence was 1,864 ms). This allowed us to embed the sentence into the background noise at a random point while maintaining a constant total duration across the set of stimuli. The start time of the target sentence within the babble noise was randomly selected for each recording from a normal distribution with a mean of 500 ms and standard deviation of 30 ms. Additional silence was appended to the end of the sentence as needed so that it had a total duration of approximately 2,500 ms (matching the duration of the noise segment). The amplitude of the noise segment was then scaled and mixed with the speech stimuli to achieve the desired SNR (based on the root-mean-square amplitudes of the signal and noise). For a given recording, the same noise sample was used for both SNRs (note that each subject only heard a given sentence at one SNR). This yielded a total of 400 sound files. The stimuli are available in the Supporting Information.

**Fig 1:**
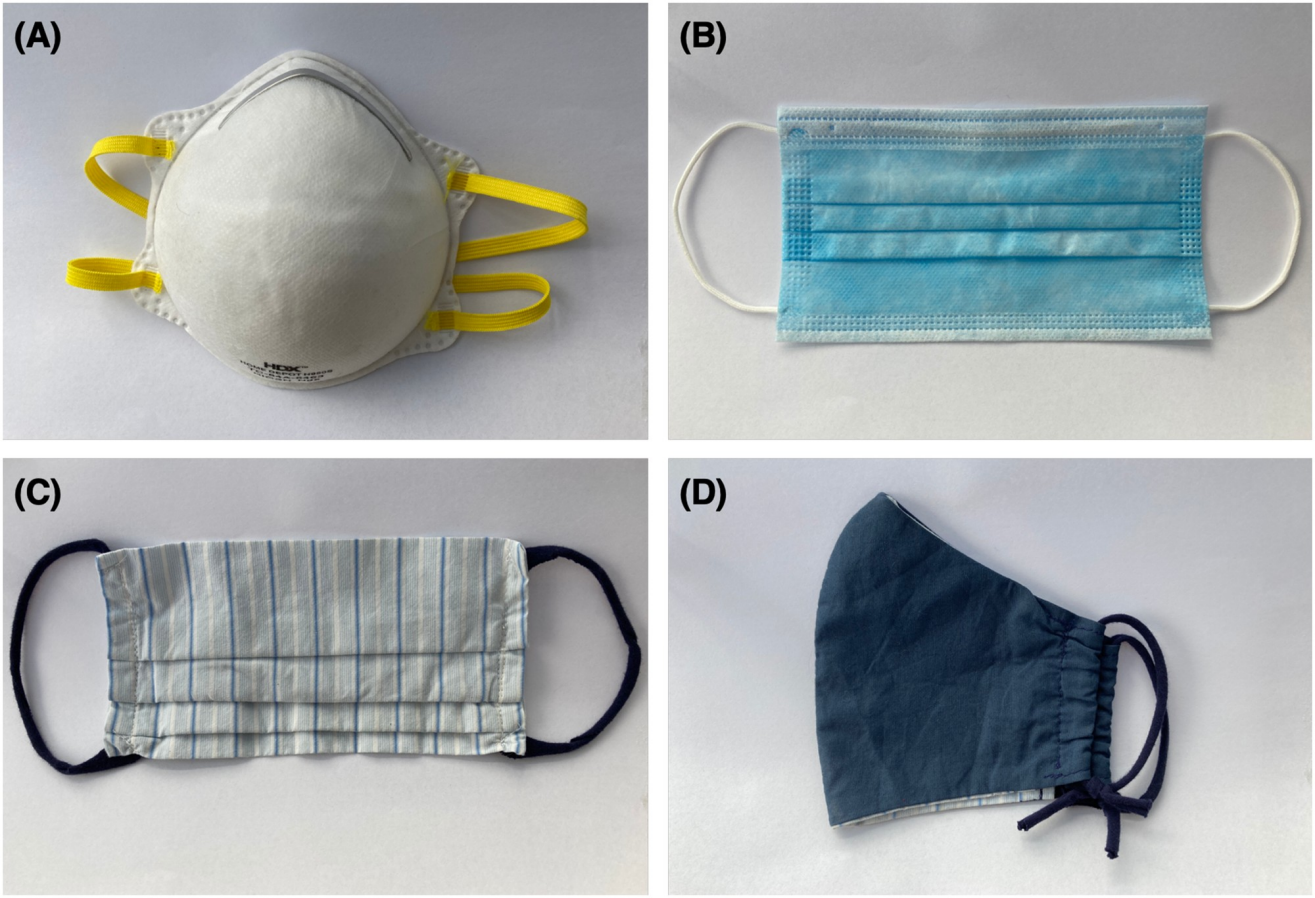
Masks used in the experiment. **(A)** N95 respirator, **(B)** surgical mask, **(C)** pleated cloth mask, **(D)** fitted cloth mask.

Four different masks were worn by each of the talkers when recording the stimuli; each talker fitted the mask themselves so that it was comfortable and similar to how they would wear the mask in everyday settings. For both talkers, the surgical mask and two fabric masks would sometimes contact the lips when speaking; this was not apparent while wearing the N95 respirator. The surgical mask was a disposable mask attached using elastic ear bands. The N95 respirator (NIOSH approval number: 84A-5463) attached using two elastic bands over the back of the head. The two cloth masks were homemade masks sewn by the second author from cotton fabric. The fitted cloth mask had two layers and was sewn from four pieces of fabric with a vertical center seam. The pleated cloth mask had two layers and was sewn from a single piece of fabric. Both masks attached behind the ears with stretch-fabric loops. The thickness of each mask was measured with a digital caliper. The surgical mask was 0.3 mm thick, the N95 respirator was 0.7 mm thick, the fitted cloth mask was 0.4 mm thick, and the pleated cloth mask was 0.3 mm thick. Photographs of each mask are provided in Fig 1.

### Procedure

The experiment was completed using Qualtrics’ (Provo, UT) survey platform. After providing informed consent and demographic information, participants confirmed that they were seated in a quiet environment and wearing headphones. Participants were also asked to type the brand or model of headphones they were wearing; these responses were examined by the first author to ensure that participants gave a reasonable answer (e.g., a particular brand, “over the ear”, etc.; see Toscano and Lansing [20] for a similar procedure). Next, participants listened to a 1 kHz test tone, scaled to the same average intensity as the speech sounds, and adjusted the volume to their most comfortable level. Limitations of online data collection make it difficult to determine the exact listening conditions of the participants (e.g., properties of headphones, overall sound level, etc.), which could affect listeners’ responses. Note, however, that these differences would not affect the SNR of the stimuli.

Listeners were instructed that they would hear recordings and were asked to type what they heard in a text box. Trials were presented separately on individual web pages. On each trial, the sentence automatically played when the page loaded, and listeners were asked to type the sentence they heard in the text box on the page. The next trial began when they clicked a button to continue. The page submission time (tracked by the Qualtrics survey) was used to assess response time.

Two practice trials (one recorded by each talker) with no background noise were presented first. This served to familiarize the subjects with both the task and the voices of the talkers. Practice sentences were recorded using the same procedure as the rest of the stimuli. After listening to the two practice sentences, subjects were told they would hear sentences spoken by the two talkers and asked to type what they heard. They were informed that there may be other voices in the background and that it might be difficult to understand what is being spoken, but that they should do their best and make a guess even if they are unsure.

After completing the two practice trials, subjects began the main experiment, which followed the same procedure as the practice trials. Subjects received a message when they reached the halfway point, which was also the point at which the SNR switched conditions.

### Data analysis

Subjects were excluded from analysis if they met any of the following criteria: (1) they self-reported having non-normal hearing (N = 6), (2) they made more than 50% errors in recognizing the words in the two practice sentences (N = 15), or (3) they failed to provide valid responses on at least 50% of the experimental trials (no subjects met this criterion). A total of 181 subjects were included in the final sample. Individual trials were excluded from analysis if the subject took longer than 60 seconds to respond. A total of 3,564 trials, across all subjects, were included in the final sample.

Responses were scored based on the number of words correctly recognized in each sentence. Alternate correct responses from the HINT sentence lists (e.g., “a” instead of “the”) were scored as correct also. Correct words were counted regardless of the position they appeared in the subject’s response (e.g., if the subject missed the first word in the sentence, the remaining words that they recognized correctly would still be counted). Note that making errors at earlier points in the sentence is likely to lead to errors at later points [21], though we have no reason to suspect that effects of word position would be differentially affected by the type of mask worn by the talker. Listeners responses were checked for common misspelled words (e.g., replacing “they’re” with “their”); these misspellings were fixed so that the response was scored correctly. A total of 21 misspellings were corrected.

Data were analyzed using mixed-effects logistic regression models implemented using the lme4 package [22] in R [23]. Models included fixed effects of mask type, talker, SNR, and their interactions. Random effects of subject and the 20 HINT sentences were also included. Talker and SNR were effect coded (Talker 1 = 0.5, Talker 2 = -0.5; +13 dB SNR = 0.5, +3 dB SNR = -0.5). Mask type was also effect coded, with the no-mask condition as the reference level. The random effects structure included by-subject and by-sentence random slopes for SNR, talker, and mask type, along with all two-way interactions (SNR × talker, SNR × mask, talker × mask). This was the maximal model justified by the design, as well as the maximal model justified by the data, as determined via a backward-stepping model comparison procedure [24]. To assess any significant interactions found in the omnibus model, we conducted follow-up analyses with mixed-effects models examining simple main effects for mask type collapsed across the other factors (i.e., to examine the SNR × talker interaction, for example, we fit two separate models with a fixed effect for talker, one at each level of SNR). The data file and analysis script are available in the Supporting Information.

To analyze the acoustic effects of the masks, spectra were created by first concatenating the individual sentence recordings for each mask condition and talker. For visual presentation of the spectra, the concatenated sounds were bandpass filtered from 50 to 11,025 Hz with a 5 Hz smoothing width. A fast Fourier transform (FFT) was then performed, and cepstral smoothing (300 Hz bandwidth) was applied to the resulting spectra. We also examined the difference in intensity between the no-mask condition and each of the mask conditions in octave-scale energy bands. An FFT was performed on the concatenated sounds, and the band energy of the spectra was computed in octave bands centered at 125, 250, 500, 1000, 2000, 4000, and 8000 Hz. The decibel values for each mask condition were then subtracted from the values for the no-mask condition in order to evaluate the decrease in sound intensity in each frequency range.

Figures were prepared using Praat [18] and the ggplot2 package [25] in R.

## Results

### Acoustic analysis

**Fig 2:**
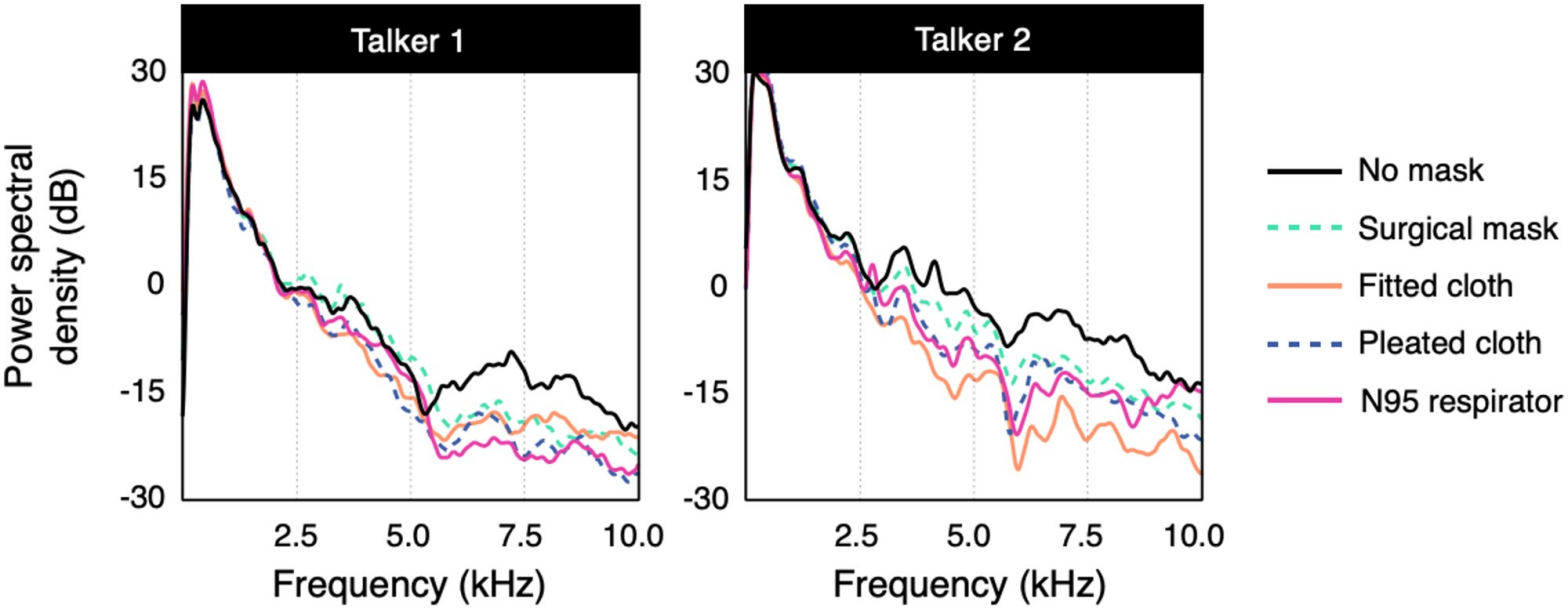
Average spectra of speech sounds produced while wearing each type of mask. The y-axis indicates the logarithmic power spectral density of the sound. Compared with the no-mask condition, face masks generally attenuated higher frequencies. They also had a greater overall effect for Talker 2, compared with Talker 1.

**Fig 3:**
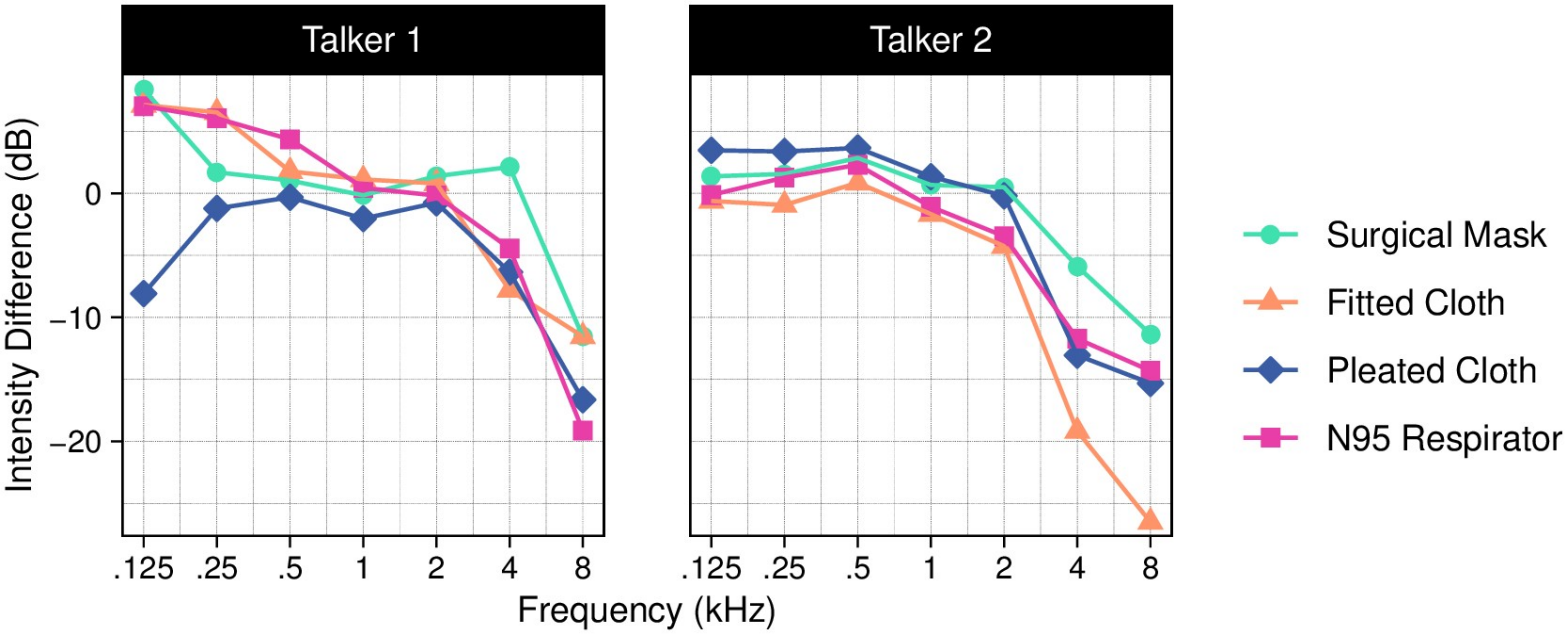
Difference in band energy between the no-mask condition and each of the four face mask conditions for sound frequencies in octave-scale bands centered at 125, 250, 500, 1000, 2000, 4000, and 8000 Hz. The acoustic effects of the masks depended on both mask type and frequency. Generally, there were small differences compared to the no-mask condition at lower frequencies and a 5–25 dB decrease in intensity at higher frequencies.

We first conducted an acoustic analysis of speech produced with each mask (Figs 2 and 3). Masks primarily attenuated sounds above 2 kHz. This effect was larger for speech produced by Talker 2, particularly for the fitted cloth mask, with a decrease in intensity of 19–27 dB relative to the no-mask condition for frequencies above 2 kHz. The difference between the two talkers could be due to several factors, such as differences in the acoustic properties of their voices, differences in how each mask fits their face, or differences in how they each produce speech while wearing a mask (thus, the differences may not be due to the sex of the talker, specifically). In contrast to the other masks, the surgical mask had a smaller effect for both talkers. Overall, these results are consistent with previous acoustic analyses of face masks [8, 9, 15].

### Speech recognition results

**Fig 4:**
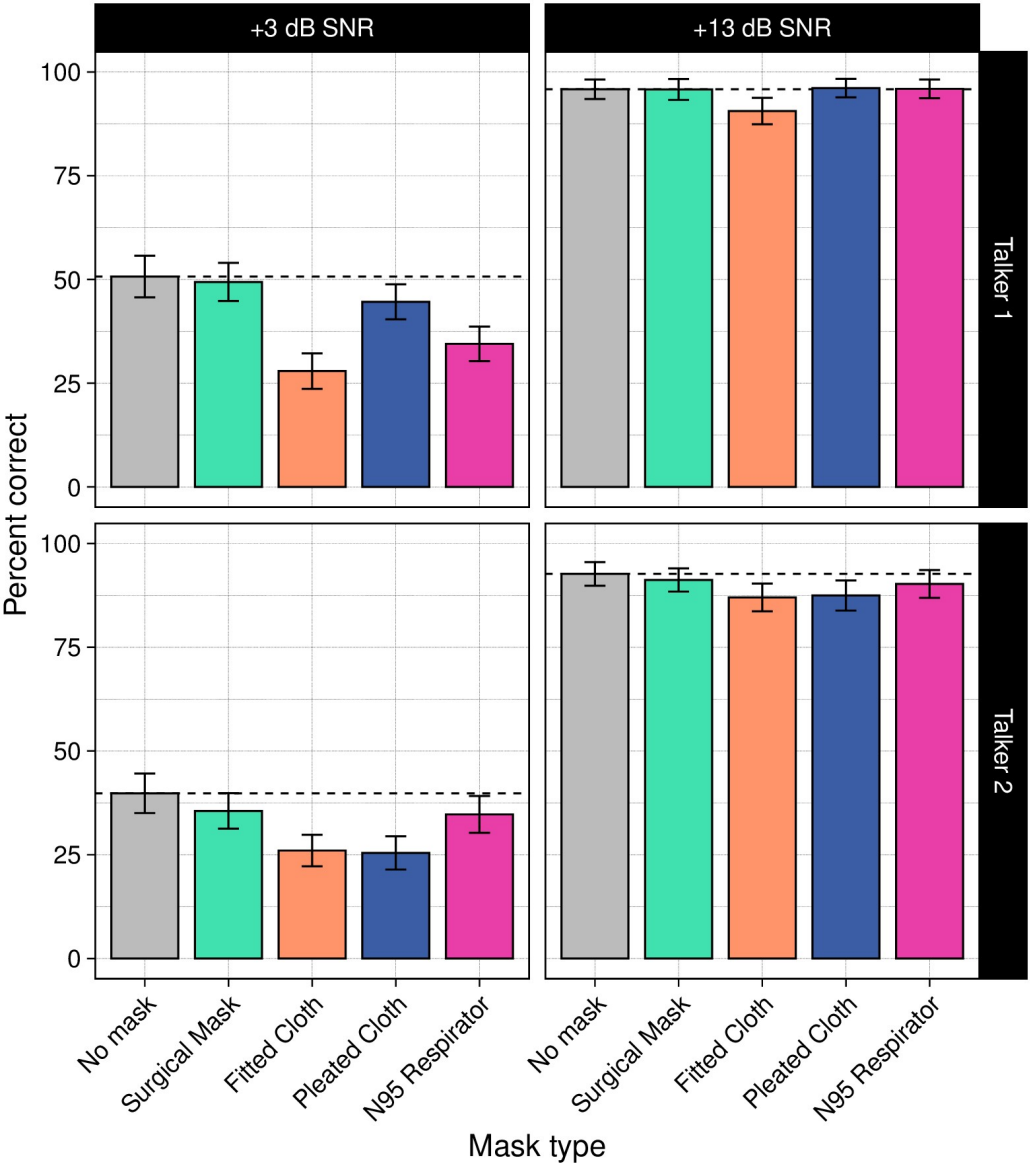
Mean percentage of words correctly recognized in the sentences as a function of mask type, signal-to-noise ratio (SNR), and talker. Horizontal dashed lines represent the mean of the no-mask condition in each panel. Overall, listeners were much more accurate at the high SNR (+13 dB) than at the low SNR (+3 dB), and they were more accurate for Talker 1 than for Talker 2. At the high SNR, only the fitted cloth mask led to poorer performance compared with the no-mask condition. At the low SNR, both cloth masks and the N95 respirator led to lower accuracy. The pleated cloth mask also caused lower accuracy for Talker 2. Errors bars represent 95% confidence intervals.

Next, we examined the impact of the masks on listeners’ speech recognition (Fig 4). Overall, as expected, listeners were much more accurate in the high SNR condition (mean accuracy: 92.2%) than in the low SNR condition (36.9%). We also observed differences as a function of talker, with higher accuracy for Talker 1 (68.3% correct overall, with 41.4% correct in the low SNR condition, and 94.5% correct in the high SNR condition) than for Talker 2 (61.3% overall; 32.4% in the low SNR condition and 89.7% in the high SNR condition). This result is consistent with previous work demonstrating that women produce more intelligible speech than men [26], though the differences observed here are not necessarily driven by talker sex (e.g., they could be due to other differences between the talkers).

Masks affected speech recognition differently depending on SNR. At the high SNR, accuracy for the no-mask condition (94.3%) was nearly the same as accuracy for speech produced with the surgical mask (93.5%) and N95 respirator (93.1%). Performance was also very high for the pleated cloth mask (91.8%) and somewhat lower for the fitted cloth mask (88.8%). For the low SNR condition, accuracy for speech produced without a mask was considerably lower (45.2%). Performance in the surgical mask condition was similar to the no-mask condition (42.4%). The other masks led to lower accuracy (N95 respirator: 34.6%; pleated cloth mask: 35.1%; fitted cloth mask: 27.0%).

These observations were validated statistically using a logistic mixed-effects model with whether or not words were correctly recognized as the dependent measure; talker, SNR, and mask type entered as fixed effects; and subject and sentence as random effects. Mask type was effect coded, with the no-mask condition as the reference level (see Method for additional details). The model revealed a main effect of SNR (*b* = 5.48, *SE* = 0.21, *z* = 25.81, *p* < 0.001), confirming that listeners performed better at the higher SNR. There was also a main effect of talker (*b* = 1.01, *SE* = 0.20, *z* = 5.13, *p* < 0.001), confirming that listeners were more accurate at recognizing speech produced by Talker 1 than Talker 2. In addition, there was a talker × SNR interaction (*b* = 0.51, *SE* = 0.25, *z* = 2.00, *p* = 0.045). Follow-up analyses revealed a main effect of talker at both SNRs (low SNR: *b* = 0.60, *SE* = 0.17, *z* = 3.50, *p* < 0.001; high SNR: *b* = 1.07, *SE* = 0.25, *z* = 4.24, *p* < 0.001).

There were also several effects of mask type. For the homemade cloth masks, there were main effects of both masks (fitted mask: *b* = −1.07, *SE* = 0.15, *z* = −7.31, *p* < 0.001; pleated mask: *b* = −0.54, *SE* = 0.18, *z* = −3.07, *p* = 0.002), demonstrating that listeners’ overall accuracy was lower than the no-mask condition. There was also a pleated mask × talker interaction (*b* = 0.79, *SE* = 0.33, *z* = 2.43, *p* = 0.015), with both talkers showing an effect in follow-up analyses (Talker 1: *b* = −0.21, *SE* = 0.10, *z* = −2.06, *p* = 0.040; Talker 2: *b* = −0.49, *SE* = 0.15, *z* = −3.29, *p* = 0.001). There were also interactions for both cloth masks with SNR (fitted mask × SNR: *b* = 0.60, *SE* = 0.30, *z* = 1.98, *p* = 0.048; pleated mask × SNR: *b* = 0.57, *SE* = 0.28, *z* = 2.08, *p* = 0.037), suggesting that the reduction in accuracy was greater at the lower SNR. Follow-up analyses revealed effects of both masks at the low SNR (fitted mask: *b* = −1.18, *SE* = 0.23, *z* = −5.20, *p* < 0.001; pleated mask: *b* = −0.67, *SE* = 0.21, *z* = −3.23, *p* = 0.001), a significant effect for the fitted mask at the high SNR (*b* = −1.25, *SE* = 0.35, *z* = −3.56, *p* < 0.001), and a marginal effect for the pleated mask at the high SNR (*b* = −0.78, *SE* = 0.41, *z* = −1.87, *p* = 0.061).

There was a main effect for the N95 respirator (*b* = −0.38, *SE* = 0.17, *z* = −2.27, *p* = 0.023), as well as an interaction with SNR (*b* = 0.83, *SE* = 0.36, *z* = 2.33, *p* = 0.020), and a three-way interaction between N95 respirator, SNR, and talker (*b* = 1.26, *SE* = 0.46, *z* = 2.75, *p* = 0.006). Follow-up analyses revealed that accuracy was lower at the low SNR for both talkers (Talker 1: *b* = −1.21, *SE* = 0.21, *z* = −5.81, *p* < 0.001; Talker 2: *b* = −0.44, *SE* = 0.22, *z* = −2.03, *p* = 0.042) but effects were non-significant at the high SNR.

There was no main effect or any interactions involving the surgical mask. Thus, accuracy was not statistically different for this mask type compared with the no-mask condition.

## Discussion

The results demonstrate that masks affect speech recognition to varying degrees depending on the talker and level of background noise. While masks produced little to no effect at the high SNR, some masks (the homemade cloth masks and N95 respirator) had a larger effect at the low SNR. In general, the acoustic dampening properties of the masks were consistent with their impact on speech recognition: the surgical mask, which produced the smallest acoustic effect also yielded the best performance; the fitted cloth mask, which attenuated sounds for Talker 2, in particular, had the poorest performance. These results are also in line with those of Bottalico et al. [17], who found poorer speech recognition performance for cloth masks in simulated classroom environments.

Note that other differences between the two talkers, beyond those captured in the spectral analysis, could have affected their intelligibility. For instance, one talker may have made certain phonetic contrasts more distinct, either in general or when wearing the masks, which could produce a compensatory effect for decreases in acoustic transmission caused by the mask (cf. [16]). Note also that the low SNR condition represents an extremely difficult listening environment—participants were only 45.2% accurate even for speech produced without a mask at this SNR. Thus, the high SNR condition is more likely to reflect situations encountered in everyday life; if high levels of background noise are anticipated, the results suggest that the surgical mask may be preferable for speech communication.

One limitation of the current study is that the results only provide data for this specific context, namely, recognizing spoken sentences in noise immediately after presentation. In addition, sentences were normalized to have the same average intensity, which partially offsets the acoustic attenuation of the masks. This may have led to better performance than might be expected when wearing a mask during face-to-face communication. However, talkers might also compensate for effects of masks in real-world settings. Indeed, recent work suggests that for certain speaking styles, such as clear speech, listeners are more accurate at recognizing speech produced with a mask than without a mask [27].

Sentences were also recorded at relatively close distances, which differs from situations encountered by listeners during the COVID-19 pandemic, where social distancing measures may also be in place. Because acoustic dampening and directivity effects of masks vary for sounds recorded at greater distances [10], masks might have different effects on speech recognition depending on the distance from the listener. Bottalico et al. [17] found similar effects to those reported here for speech stimuli created to simulate classroom environments, and Magee et al. [16] found similar acoustic effects of masks recorded from both a head-mounted microphone and tabletop microphone at a distance of approximately 5 feet. Together, these results suggest that face masks may have similar effects for speech heard at greater distances, but additional research is needed.

In addition, all masks investigated in this study are affected by the loss of visual speech information, which is important for accurate recognition [28] and for recall from memory [29]. Different types of masks also have different effects on audiovisual speech recognition [30]. However, accuracy was still very high even without visual information in the high SNR condition (94.3% correct for the no-mask condition). Future work should investigate whether the loss of visual information interacts with the loss of auditory information. Moreover, while the current study considers the effects of masks on speech recognition for listeners with normal hearing, masks have a greater impact on speech recognition for listeners with hearing loss [31] and visual information may be more important [11]. The effects of different masks must be further evaluated to determine how mask type, presence of visual information, and level of background noise might affect listeners with hearing loss. Finally, future work aimed at investigating listeners’ perception of specific speech sounds (e.g., differences between fricatives that are signaled by high-frequency acoustic cues; [32]) would further inform our understanding of how masks affect speech recognition.

In conclusion, the results demonstrate that, in low levels of background noise for the context examined here (i.e., recognizing spoken sentences immediately after presentation), face masks have only a small effect relative to speech produced without a mask, and some masks have no effect. In high levels of background noise, the effects of different types of masks become more apparent—the homemade cloth masks and N95 respirator had the largest impacts on speech recognition in this condition, while the surgical mask showed no effect.

## Supporting information

S1 File Analysis script. (R)

S2 File Data file. (CSV)

S3 File Additional analyses. (PDF)

S4 File Stimuli part 1. (ZIP)

S5 File Stimuli part 2. (ZIP)
