# Supplementary material for: Effects of face masks on speech recognition in multi-talker babble noise
Source: PLoS One. 2021 Feb 24;16(2):e0246842. doi: 10.1371/journal.pone.0246842 (PMC7904190; doi:10.1371/journal.pone.0246842)
Supplement: S3 File — (PDF) [file pone.0246842.s003.pdf]

Supporting Information for

Effects of face masks on speech recognition in multi-talker babble noise

Joseph C. Toscano & Cheyenne M. Toscano

Additional Acoustic Analyses

To confirm that the acoustic effects from wearing the different types of masks remained relatively stable across recording conditions, we re-recorded the original sentences produced by both talkers in a sound-attenuated booth. We also recorded the sentences twice, reversing the order in which the masks were worn for each set of recordings, in order to counterbalance any effects of vocal fatigue. Overall, the new recordings showed a pattern of effects similar to the original stimuli. In general, masks attenuated higher frequency components of the speech signal (Fig. S1), consistent with prior work. Similarly, we again observed the smallest effects for the surgical mask and the largest effects for the fitted cloth mask. For the octave-scale band energy analysis (Fig. S2), we also computed the difference between the values obtained for these recordings and the corresponding values for the original stimuli. The mean difference across talker and mask conditions was 1.7 dB with a standard deviation of 5.1 dB, confirming that the differences across mask types are greater than the variability across recording conditions. These results suggest that the acoustic effects of wearing a mask are consistent across recording environments and across the order in which the masks are worn during recording.

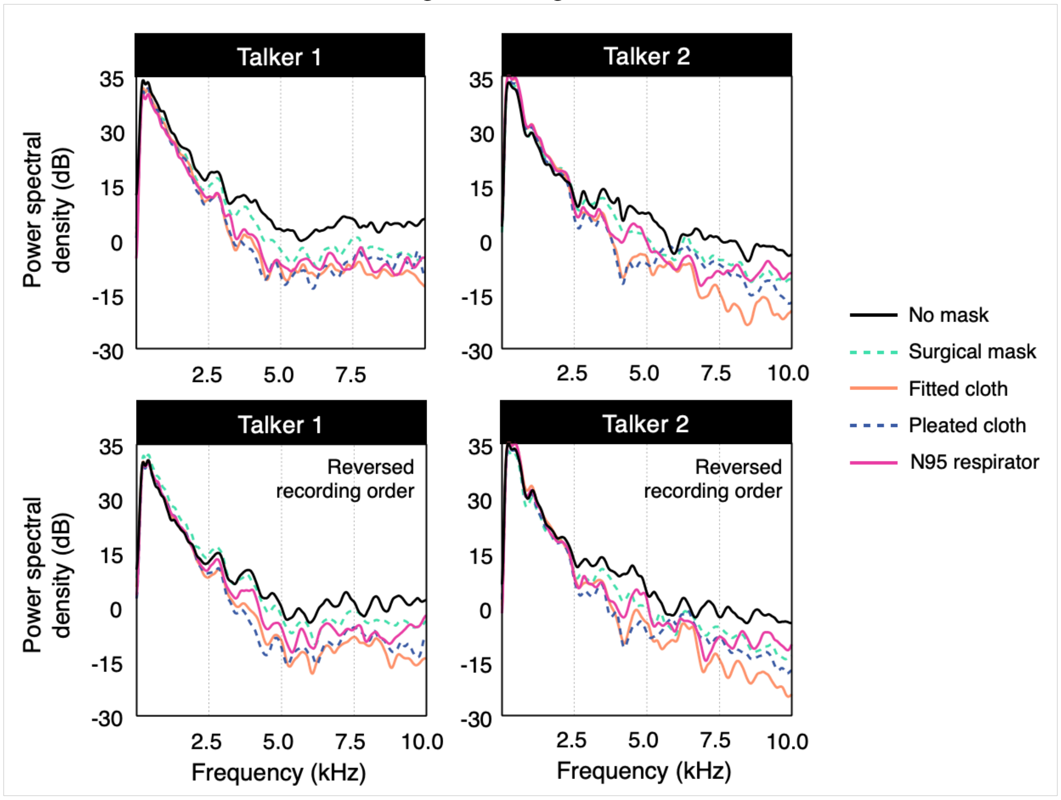

Figure S1: Average spectra for sentences recorded while wearing each type of mask (as in Fig. 2 in the main text) for recordings made in a sound-attenuated booth. The y-axis shows the logarithmic power spectral density. The bottom panels show the spectra for recordings made in the opposite order from those in the top panel.

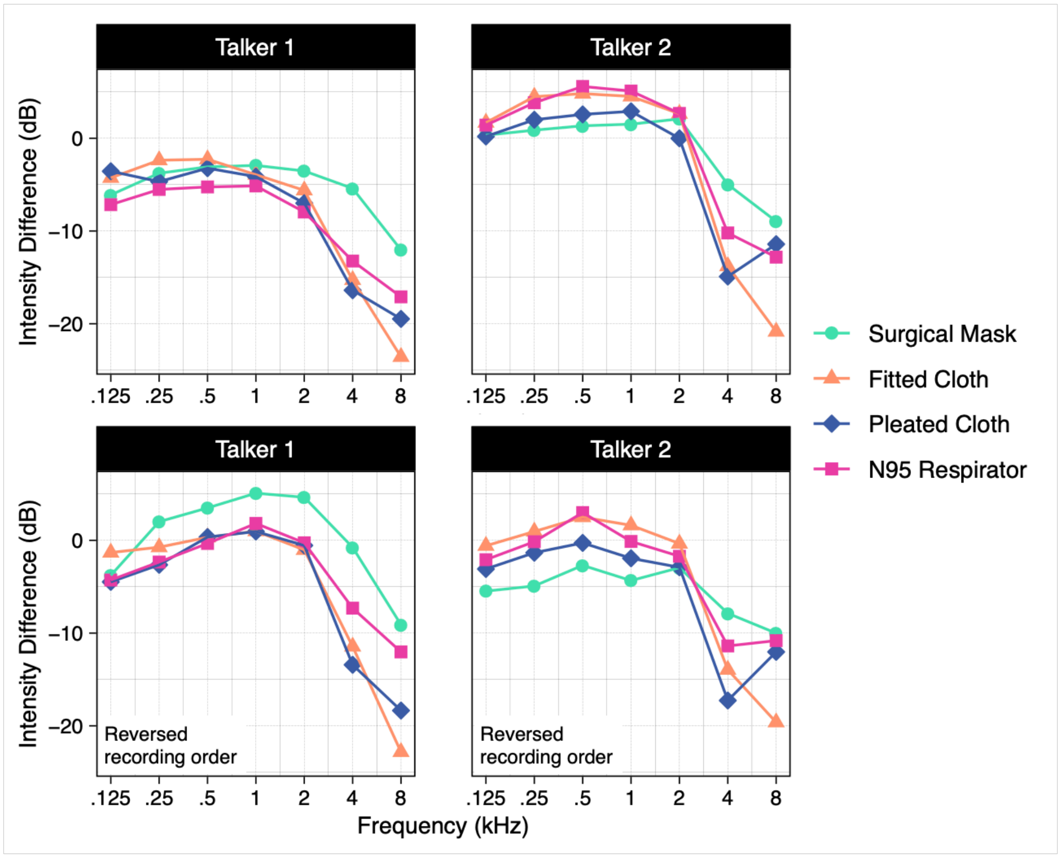

Figure S2: Differences in decibel values relative to the no mask condition for octave-scale energy bands (as in Fig. 3 in the main text). The mean difference between these values and the corresponding values in the original stimuli is 1.7 dB.
